# Derivation of Transgene‐Free Rat Induced Pluripotent Stem Cells Approximating the Quality of Embryonic Stem Cells

**DOI:** 10.5966/sctm.2015-0390

**Published:** 2016-09-13

**Authors:** Shuping Li, He Lan, Hongsheng Men, Yuanyuan Wu, Ning Li, Mario R. Capecchi, Elizabeth C. Bryda, Sen Wu

**Affiliations:** ^1^ State Key Laboratory of Agrobiotechnology, College of Biological Sciences, China Agricultural University, Beijing, People's Republic of China; ^2^ Rat Resource and Research Center, Veterinary Pathobiology, University of Missouri, Columbia, Missouri, USA; ^3^ Department of Human Genetics, University of Utah School of Medicine, Salt Lake City, Utah, USA

**Keywords:** Rat, Induced pluripotent stem cells, Embryonic stem cells, Transgene‐free

## Abstract

Although a variety of reprogramming strategies have been reported to create transgene‐free induced pluripotent stem (iPS) cells from differentiated cell sources, a fundamental question still remains: Can we generate safe iPS cells that have the full spectrum of features of corresponding embryonic stem (ES) cells? Studies in transgene‐free mouse iPS cells have indicated a positive answer to this question. However, the reality is that no other species have a derived transgene‐free iPS cell line that can truly mimic ES cell quality. Specifically, critical data for chimera formation and germline transmission are generally lacking. To date, the rat is the only species, other than the mouse, that has commonly recognized authentic ES cells that can be used for direct comparison with measure features of iPS cells. To help find the underlying reasons of the current inability to derive germline‐competent ES/iPS cells in nonrodent animals, we first used optimized culture conditions to isolate and establish rat ES cell lines and demonstrated they are fully competent for chimeric formation and germline transmission. We then used episomal vectors bearing eight reprogramming genes to improve rat iPS (riPS) cell generation from Sprague‐Dawley rat embryonic fibroblasts. The obtained transgene‐free riPS cells exhibit the typical characteristics of pluripotent stem cells; moreover, they are amenable to subsequent genetic modification by homologous recombination. Although they can contribute significantly to chimeric formation, no germline transmission has been achieved. Although this partial success in achieving competency is encouraging, it suggests that more efforts are still needed to derive ground‐state riPS cells. Stem Cells Translational Medicine *2017;6:340–351*

**Significance Statement**

To date, no species other than the mouse have derived transgene‐free induced pluripotent stem (iPS) cells that can truly mimic ES cell quality. In the current study, episomal vectors were used to obtain rat transgene‐free iPS cells, which contributed to chimeric formation. This research contributes to a better understanding of the technical limitations in generating germline‐competent embryonic stem (ES)/iPS cells and provides valuable clues for overcoming the difficulties of acquiring ground‐state iPS/ES cells in other species, including humans.

## Introduction

Mouse embryonic stem (ES) cells were first successfully established in 1981, and their usefulness was best exemplified in the subsequently developed mouse knockout technology (gene targeting) that has revolutionized biomedical research for the past 3 decades 1, 2, 3. Encouraged by this, since the early 1980s, researchers have spent great efforts trying to establish ES cells for other species including human 4, 5, 6, 7, 8, 9. However, success is limited; only mice and rats have produced authentic ES cells, with competency for germline transmission 10, 11, 12. The status of ES cells from other species including humans is still controversial, as critical data for chimera formation and germline transmission are not available for these ES cells 13, 14.

In 2006, the logic for conventional ES cell derivation was challenged when Yamanaka successfully used four transcription factors to convert differentiated mouse somatic cells into pluripotent stem cells, named induced pluripotent stem (iPS) cells, which resemble ES cells in contributing to the three germ layers and generating germline‐competent mice 15, 16. This new strategy was quickly used to generate iPS cells from a range of species, including rat 17, 18, pig 19, 20, 21, monkey 22, sheep 23, cow 24, dog 25, rabbit 26, equine 27, and even human disease‐specific cells 28, 29, 30. It became clear that the initial strategies depending on viral systems resulted in random exogenous gene integration and safety concerns 15, 31. Recent efforts focused on developing technologies to derive transgene‐free iPS cells, with the aim of obtaining cells that are both safe and of high quality (i.e., germline competency or equivalent) 32, 33, 34, 35, 36, 37. Most of these iPS cells demonstrate some ES cell‐specific features such as typical morphology, expression of specific cell surface markers, and differentiation potential in vitro and in vivo. It appeared that the availability of iPS cells solved the lack of ES cells in other species. However, a fundamental question still remains: Are iPS cells really equivalent to ES cells? In stark contrast to people's misconceptions, no germline‐competent transgene‐free iPS cell line has been established in any species other than mice.

The rat is the only other species besides the mouse that has commonly recognized ground‐state ES cells. This provides an excellent opportunity for direct comparison with iPS cells to better understand technical limitations in generating germline‐competent ES/iPS cells. Current rat iPS (riPS) cell generation relies on techniques that lead to permanent integration of transcription factors in the iPS cell genome 17, 18, 38, 39, 40, 41, 42, 43, 44, 45, 46. Integration‐free riPS cells have also been attempted by *piggyBac* transposon system 47, yet the competency of these cells was not determined.

In the current study, we described the generation of transgene‐free riPS cells with qualities approximating ES cells. Using episomal vectors containing eight transcription factors, we exploited hypoxic culture conditions combined with optimized culture medium to facilitate the generation of riPS cells. These riPS cells exhibit the typical expression of pluripotent markers and differentiation potential. In particular, we found the riPS cells were readily amendable to robust and accurate gene modification by homologous recombination, a quality found in ES cells. The riPS cells contributed to a high percentage of chimerism in chimeras generated by blastocyst injection. Unfortunately, no germline transmission has been observed through extensive breeding. Our results suggest that current reprogramming strategies, not culture conditions, are the main obstacles for obtaining authentic ground‐state riPS cells. Lessons learned from riPS cells are critical for the advancement of the entire iPS and ES cell fields.

## Materials and Methods

### Animals

Sprague‐Dawley rats were purchased from Charles River Laboratories (Wilmington, MA, http://www.criver.com). Male Dark Agouti (DA) rats were purchased from Shanghai Laboratory Animal Research Center (Shanghai, China, http://english.sibs.cas.cn/rs/fs/ShanghaiLaboratoryAnimalCenterCAS). All procedures of cell culture or reproductive studies using animals were approved by Laboratory Animal Care and Use Committee of China Agricultural University.

### Cell Culture

Rat embryonic fibroblasts and mouse embryonic fibroblast (MEF) feeders were cultured in MEF medium consisting of Dulbecco's modified Eagle's medium (DMEM; Thermo Fisher Scientific, Waltham, MA, https://www.thermofisher.com) supplemented with 1× nonessential amino acids (Thermo Fisher), 1× GlutaMAX (Thermo Fisher), 1× penicillin‐streptomycin (Thermo Fisher), and 1× sodium pyruvate solution (Thermo Fisher). Obtained riPS cells were maintained on Co^60^‐radiated MEF feeders in 3i/Lif medium (N2B27 medium supplemented with 1 μM PD0325901 [Selleck Chemicals, Houston, TX, http://www.selleckchem.com], 3 μM CHIR99021 [Selleck], 0.5 μM A83‐01 [Tocris, San Diego, CA, http://www.tocris.com], 100× penicillin‐streptomycin [Thermo Fisher], 0.1 mM 2‐mercaptoethanol [Sigma‐Aldrich, St. Louis, MO, http://www.sigmaaldrich.com], and 1,000 units/ml rat Lif [ESGRO, Chemicon, Millipore, Bedford, MA, http://www.millipore.com]). N2B27 medium consisted of a mixture of 500 ml of DMEM/F12 medium (Thermo Fisher), 500 ml of Neurobasal medium (Thermo Fisher), 5 ml of N2 supplement (Thermo Fisher), and 10 ml of B‐27 supplement (Thermo Fisher).

### Establishment of Rat ES Cells From Blastocysts

Sprague‐Dawley rat embryos at blastocyst stage (4.5 days pregnant) were flushed out using *m*‐RECM‐1‐HEPES medium 48 and cultured in 3i medium for 5 days on MEF feeders. Subsequently, outgrowths were dissociated into single cells with Accutase (Sigma‐Aldrich) and seeded onto new MEF feeders. Colonies that appeared were passaged again and expanded until stable rat ES cell lines were generated.

### Generation of riPS Cells From Rat Embryonic Fibroblasts

Plasmids carrying reprogramming factors were transfected into rat embryonic fibroblasts by electroporation using Lonza Amaxa Nucleofector (Lonza, Cologne, Germany, http://www.lonza.com). Transfection was conducted according to manufacturer's instruction. The optimal nucleofection conditions for fibroblasts were as follows: A‐024 program with 3‐μg plasmids per 10^6^ cells. The fibroblasts were cultured in feeder medium without Lif on MEF feeder layers prepared 1 day before nucleofection. Twenty‐four hours after nucleofection, transfected fibroblasts were subjected to positive selection using G418 (400 μg/ml; EMD Chemicals, Inc., San Diego, CA, http://www.emdchemicals.com) in serum containing medium supplemented with rat Lif for 5 days. Established riPS cell lines were cultured in 3i/Lif or 2i/Lif medium for subsequent passaging and propagation.

### Derivation of Transgene‐Free riPS Cells

The primary pMaster12‐ and pMaster22‐derived riPS cells were grown in the presence of 0.3 μM 1‐(2‐deoxy‐2‐fluoro‐β‐D‐arabinofuranosyl)‐5‐iodoracil (FIAU) at passage 2 (20,000 cells) to obtain transgene‐free subclones. The FIAU‐insensitive subclones were picked and passaged once before performing G418 selection (400 μg/ml) in 3i/Lif medium. After double drug screening, clones showing a FIAU‐insensitive and G418‐sensitive phenotype, indicating absence of *HSVtk* and *neo* genes, were further tested by polymerase chain reaction (PCR) to confirm riPS cells were transgene free.

### Genomic PCR and Quantitative Real‐Time PCR

Genomic DNA was extracted from riPS cells according to protocols described previously 49. Total RNA was extracted by TRIzol reagent (Thermo Fisher) according to the manufacturer's instruction. cDNA was synthesized from 1 μg of total RNA using QuantiTect Reverse Transcription kit (Qiagen, Hilden, Germany, http://www.qiagen.com). Before cDNA synthesis, the purified RNA sample is briefly incubated in the DNase containing gDNA Wipeout Buffer at 42°C for 2 minutes to effectively remove contaminating genomic DNA. Quantitative real‐time PCR (q‐PCR) analysis was performed using SYBR Green Real‐time PCR Master Mix (Roche, Basel, Switzerland, http://www.roche.com) in triplicate. Gene expression levels were normalized to expression of the house‐keeping gene glyceraldehyde‐3‐phosphate dehydrogenase (*Gapdh*). Relative expression of each gene was quantified from threshold cycles for amplification using the ∆∆C_T_ method. PCR primers are listed in supplemental online Table 8.

### Immunofluorescence Staining

For immunofluorescence assays, cells were fixed in 4% (wt/vol) paraformaldehyde for 30 minutes at room temperature and incubated in blocking buffer (5% [vol/vol] goat serum and 1% [wt/vol] bovine serum albumin) for 1 hour with or without 0.2% triton X‐100 (Sigma‐Aldrich) for nuclear staining and membrane staining, respectively. Cells were then incubated with primary antibody overnight at 4°C, followed by secondary antibody hybridization conducted in the dark at recommended concentrations (Thermo Fisher). The primary antibodies were as follows: anti‐Oct4 (mouse IgG2b, 1:100; Santa Cruz Biotechnology, Santa Cruz, CA, http://www.scbt.com), anti‐Sox2 (goat polyclonal, 1:500; Santa Cruz), anti‐SSEA‐1 (mouse IgM, 1:500; Abcam, Cambridge, MA, http://www.abcam.com), anti‐NANOG (mouse IgM, 1:500, Abcam), anti‐GATA4 (rabbit polyclonal, 1:500; Abcam), anti‐α‐smooth muscle actin (rabbit polyclonal, 1:500; Abcam), and antinestin (chicken polyclonal, 1:500; Abcam). Nuclei were counterstained using 4′,6‐diamidino‐2‐phenylindole (Roche) at a concentration of 0.1 μg.

### Teratoma Formation and Histological Examination

Approximately 5 × 10^6^ riPS cells were resuspended in phosphate‐buffered saline and injected subcutaneously into a 5‐week‐old severe combined immunodeficient mouse purchased from Charles River Laboratories; 6 weeks later, the teratoma were removed and then embedded in paraffin wax for histological processing. Slides with teratoma sections were stained with hematoxylin and eosin (H&E) and examined under a microscope.

### Generation of Chimera by Microinjection

On the day of microinjection, riPS cells at passage 11 to 16 were disassociated into single cell suspension by Accutase (Sigma‐Aldrich). After pelleting by centrifugation, the cells were resuspended in 3i/Lif supplemented with 20 mM HEPES. Fifteen to 20 riPS cells were injected into DA × Sprague‐Dawley hybrid blastocysts. After brief culture in mR1CM after injection, approximately 30 blastocysts were surgically transferred into the uterine horns of day‐3.5 pseudopregnant Sprague‐Dawley female rats (approximately 15 blastocysts per uterine horn).

### Construction of *Leptin* Gene Targeting Vector

The targeting vector was designed and constructed using a previously reported protocol 49, 50. The homologous arms in the targeting vector were amplified using Pfu UltraII Fusion HS DNA Polymerase (Stratagene, La Jolla, CA, http://www.stratagene.com) with the Sprague‐Dawley rat genome as a template.

### Electroporation of riPS Cells

The *Leptin* targeting vector containing a *neo*/*HSVtk* double selection cassette was linearized with *Swa* I. Approximately 2 × 10^6^ riPS cells were electroporated with 6 μg linearized targeting vector using the Lonza Amaxa Nucleofector (Lonza) program B‐016. Electroporated cells were plated into 10‐cm culture dish containing 3i/Lif medium and irradiated feeders. G418 (400 μg/ml) and FIAU (0.3 μM) selection were initiated 24 hours postelectroporation and surviving colonies were picked and propagated for additional characterization.

### Southern Blot Analysis

Southern blot analysis was carried out using a protocol described previously with slight modifications 49. Briefly, genomic DNA (10 μg) was digested with *Kpn* I enzyme for 24 hours. Probes were prepared by PCR amplification with PCR DIG Probe Synthesis Kit (Roche). Primers used for probe amplification are as follows: 5ʹ forward GCATTTCAAGCTCTGCCCAG with reverse CACTGAGTTCCCTGAGACCC and 3ʹ forward GTAAGGGAGGGGCTGGACTCCCAA with reverse CTGAGATGCAGTGTCTCTTTCAG.

## Results

### Establishment of Rat ES Cell Lines Using Improved Culture Conditions

To provide a direct comparison for riPS cells, we first tried to establish ES cell lines from Sprague‐Dawley rats, which were proved for the derivation of germline‐competent ES cells 11, 51. Considering the germline‐competent rat ES cells were established in 2i medium 11 and subsequently transforming growth factor‐β (TGF‐β) inhibitor A83‐01 was applied to facilitate rat ES cell establishment 52, we tried to isolate rat ES cells with serum‐free N2B27 medium supplemented with CHIR99021, PD0325901, and A83‐01 (3i/Lif medium).

**Figure 1:**
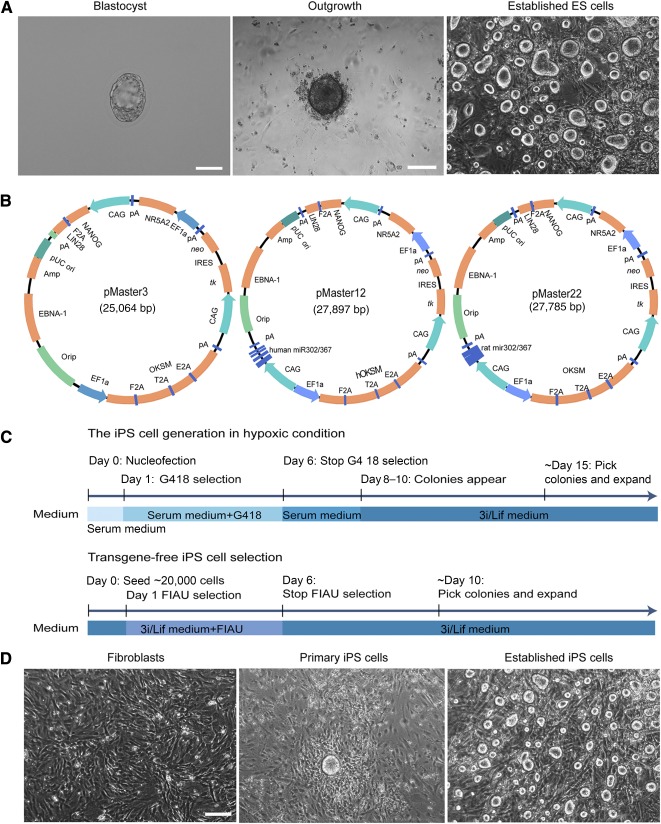
Establishment of riPS cells and ES cells. **(A):** Derivation of rat ES cells from blastocyst outgrowth. We flushed E4.5 blastocysts from the uterus of pregnant Sprague Dawley rats (left). Scale bar = 100 μm. The blastocysts were plated on feeders in the 3i/Lif medium until the primary outgrowth attached at day 4. ES cells were established from blastocyst outgrowth after three passages and showed typical ES cell morphology. Scale bar = 200 μm. **(B):** Schematic diagram of the EBNA/oriP‐based reprogramming vector. Plasmid pMaster3 contains 7 human transcription factor genes of *OCT4/POU5f1*, *SOX2*, *KLF4*, *C‐MYC*, *NANOG*, *LIN28*, and *NR5A2* for reprogramming and two drug resistance genes, *neo* and *HSVtk*, for positive/negative selection. Plasmid pMaster12 and pMaster22 with additional human and rat *miR‐302/367* gene cluster, respectively. **(C):** Flowchart of transgene‐free riPS cell derivation. Rat fibroblast cells were seeded on mouse embryonic fibroblast feeders and cultured in serum medium after electrotransfection with pMaster3/pMaster12/pMaster22, followed by G418 drug selection for 5 days to ensure successful plasmid transfection. After the eighth day, 3i/Lif medium was used for riPS cell maintenance and primary clones were picked and propagated. To obtain transgene‐free subclones, we plated 20,000 cells into a 10‐cm dish to carry out FIAU negative selection to exclude cells carrying transgenes after the second day. Clones that passed negative selection were picked and expanded for further verification. G418, geneticin, a derivative of gentamycin; serum medium, Dulbecco's modified Eagle's medium supplemented with fetal bovine serum and Lif; and 3i/Lif medium, N2B27 based serum‐free medium with additional CHIR99021, PD0325901, A83‐01, and Lif. **(D):** Cell morphology during different reprogramming stages. Rat embryonic fibroblasts at passage 1 were used for reprogramming. Primary riPS cells have rat ES‐cell like morphology at day 10 after electrotransfection under hypoxic culture conditions in 3i/Lif medium. The established iPS clones showed round and smooth border morphology, which is a typical characteristic of ES cells. Scale bar = 200 μm. Abbreviations: ES, embryonic stem; FIAU, 1‐(2‐deoxy‐2‐fluoro‐1‐D‐arabinofuranosyl)‐5‐iodoracil; iPS, induced pluripotent stem; Lif, leukemia inhibitory factor; riPS, rat induced pluripotent stem.

First, blastocysts collected from 4.5‐day‐pregnant Sprague‐Dawley rats were cultured in 3i/Lif medium until the inner cell mass‐derived outgrowth appeared and expanded (Fig. 1A). The primary outgrowths were dissociated with Accutase into single cells and transferred onto fresh feeders. After continuous culture of five passages, stable cell lines with typical morphology of ES cells were established (Fig. 1A). Using 3i/Lif medium, we established ES cells at significantly higher efficiency (60%, 15 of 25) than 2i/Lif medium (20%, 2 of 10) (supplemental online Table 1), suggesting that addition of A83‐01 is beneficial for ES cell derivation and maintenance.

### Failed Reprogramming With Episomal Vectors Using Conventional Culture Conditions

To derive high‐quality transgene‐free riPS cells, we used the nonintegrative episomal system that had been successfully used in mice to generate transgene‐free iPS cells with competency for efficient germline transmission 53. Three episomal vectors including two previously reported, pMaster3 and pMaster12, were used in this study (Fig. 1B). The initial reprogramming of mouse and human somatic cells into iPS cells were separately achieved by expressing combinations of transcription factors including *OCT4*, *SOX2*, *KLF4*, *C‐MYC* and *OCT4*, *SOX2*, *NANOG*, and *LIN28* 16, 54. It has been reported that *Nr5A2* (nuclear receptor subfamily 5, group A, member 2) can regulate OCT4 expression 55 and replace *Oct4* to accomplish the reprogramming of mouse somatic cells into iPS cells 56. In addition, *Nr5A2* is a potent inducer of epiblast stem cells (EpiSCs) reprogramming into ground‐state pluripotency 57. The pMaster3 vector was constructed to contain all of the previously mentioned seven factors to facilitate reprogramming. Because overexpression of *miR‐302* can enhance reprogramming of somatic cells into iPS cells 58, 59, we included human and rat *miR‐302/367* gene clusters in the pMaster12 and pMaster22 vectors, respectively, in addition to genes on pMaster3 (supplemental online Table 2). All three vectors contain the *neo* resistance gene to ensure successful transfection and the negative selection gene *HSVtk* to facilitate the transgene removal process. The thymidine kinase (*tk*) gene is one of the most widely used negative selection marker in gene targeting experiments 60. FIAU, a nucleoside analog of ganciclovir, was a substrate for thymidine kinase 61. In the presence of the *HSVtk* gene, FIAU is converted into toxic compounds and consequently cells selected against. In the absence of the *tk* gene, cells are resistant to FIAU selection. In our study, to facilitate the generation of transgene‐free riPS cells, we introduced this negative selection cassette into the reprogramming vectors.

Next, to test the reprogramming efficiency of the three episomal plasmids on riPS generation, plasmids pMaster3, pMaster12 and pMaster22 were independently used to generate riPS cells. Twenty‐four hours after Sprague‐Dawley rat embryonic fibroblasts were separately transfected with plasmids, G418 was applied to the culture media and continued for 5 days to ensure successful transfection of the reprogramming genes. When ES‐like colonies first appeared at approximately day 14, serum medium was changed to N2B27 based 3i/Lif medium. At approximately 3 weeks post‐transfection, the appeared primary colonies were picked and expanded (supplemental online Fig. 1A). Compared with many other protocols, our transfected rat fibroblasts were not passaged after electroporation, ensuring most of the primary colonies were monoclonal. In addition, count of primary colonies was more likely to reflect the real reprogramming efficiency. Whereas plasmid pMaster3 failed to generate ES cell‐like colonies, pMaster12 was able to reprogram rat embryonic fibroblasts into ES‐like colonies at a low efficiency of approximately 0.001% (8 ± 2 per 10^6^ cells), whereas the efficiency of pMaster22 at approximately 0.002% (20 ± 2 per 10^6^ cells), suggesting that rat‐specific *miR‐302/367* gene cluster enhanced reprogramming efficiency (supplemental online Table 3). These pMaster12‐ and pMaster22‐derived primary clones were flat and tightly adhered to the culture dish (supplemental online Fig. 1B). This morphology is different from the typical domed colony morphology of rat ES cells. Twelve primary colonies reprogrammed by pMaster22 were picked and only two of them could be subsequently passaged, indicating that a majority of these clones were not well reprogrammed. In contrast to the fast self‐renewal rate of high‐quality ES cells, growth of the two established clones was relatively slow and they needed to be passaged at 5‐to 6‐day intervals. It was very difficult to generate riPS cells with conventional culture methods and the few riPS cells established were of slow growth that needed to improve. This suggested that better reprogramming methods suited for rats would be needed to generate transgene‐free riPS cells.

### Successful Generation of Transgene‐Free riPS Cells With Optimized Reprogramming Conditions

To improve reprogramming efficiency with episomal vectors, optimized ES culture conditions that combined a hypoxic culture environment (5% O_2_) 62 were used. The three plasmids also were independently used to generate riPS cells. The strategy used for generating riPS cells in hypoxic conditions was the same as for normoxic condition except that the whole procedure of reprogramming was performed in hypoxic condition. We found at these new conditions, all three plasmids were able to independently reprogram rat embryonic fibroblasts into iPS cell lines, but at different efficiencies. For pMaster3, the reprogramming efficiency was very low (4 ± 1 per 10^6^ cells) and only half the isolated clones survived repeated passages to riPS cell line generation. The reprogramming efficiencies of pMaster12 and pMaster22 were increased 10‐fold (96 ± 4 per 10^6^ cells; 198 ± 2 per 10^6^ cells) compared with reprogramming efficiencies under normoxic culture conditions (supplemental online Table 3). In addition, under hypoxic conditions, the time from transfection to the formation of primary clones was reduced to 10 days (Fig. 1C, 1D), 5 to 7 days shorter than reprogramming under normoxic conditions. Importantly, unlike primary clones derived under normoxic condition, these clones exhibited round domed morphology, similar to authentic rat ES cells (Fig. 1A, 1D). Reprogramming by pMaster22 with the rat‐specific *miR‐302/367* gene cluster produced two to three times more primary clones than pMaster12 that contains the human *miR‐302/367* gene cluster. The reprogramming process was repeated multiple times and details are given in supplemental online Table 4. Hypoxic culture conditions play a key role in riPS cell generation and the rat‐specific *miR‐302/367* gene cluster significantly promotes reprogramming.

**Figure 2:**
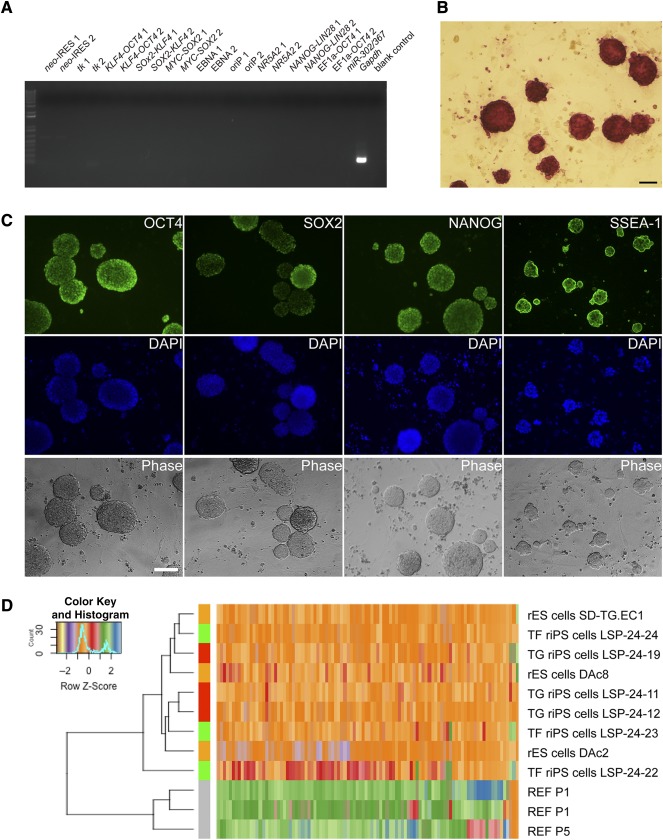
Confirmation and characterization of transgene‐free riPS cells. **(A):** Confirmation of transgene‐free riPS cells by polymerase chain reaction (PCR) in a representative clone LSP‐24‐24. No vector fragment was detected by PCR using 21 primer pairs covering the entire vector whereas the endogenous *Gapdh* gene was detected. For each fragment of the vector (e.g., *neo*‐IRES), we used two pairs of primers (e.g., *neo*‐IRES 1, *neo*‐IRES 2). PCR with H_2_O template was used as blank control. **(B):** Alkaline phosphatase staining of riPS cells (LSP‐24‐24, passage 25). Scale bar = 100 μm. **(C):** Immunofluorescence staining of transgene‐free riPS cell line LSP24‐24. Positive staining for OCT4, SOX2, NANOG, and SSEA‐1, indicating transgene‐free riPS cells maintain expression of pluripotent markers. Nuclei were stained with DAPI. Scale bar = 200 μm. **(D)**: Heat‐map and unsupervised hierarchical clustering of REFs, transgene‐free riPS cells (LSP‐24‐22, LSP‐24‐23, LSP‐24‐24), transgene‐intact riPS cells (LSP‐24‐11, LSP‐24‐12, LSP‐24‐19), and rES cells (SD‐Tg.EC1, DAc2, DAc8) 11, 51. Abbreviations: DAPI, 4′,6‐diamidino‐2‐phenylindole; PCR, polymerase chain reaction; rES, rat embryonic stem; TF, transgene free; TG, transgene intact; REF, rat embryonic fibroblast; riPS, rat induced pluripotent stem.

To obtain transgene‐free riPS cells, FIAU selection was used to facilitate transgene‐free iPS generation. Because thymidine kinase can convert FIAU substrate into toxic metabolites, cells still harboring the original pMaster vectors with the *tk* gene were selectively killed 49, 60. Approximately 20,000 riPS cells were seeded in a 10‐cm dish to apply the FIAU selection, and approximately 10 colonies could grow up. These survived subclones were picked and expanded for confirmation of exogenous gene removal. The results of PCR analysis showed that, of 36 FIAU‐selected clones cultured in 2i/Lif medium, 17 (47.2%) clones were free of transgenes; 3 (8.3%) clones contained a complete set of intact transgenes; 16 (44.4%) clones contained 1 or more of the residual transgenes including *OCT4*, *SOX2*, *KLF4*, *NANOG*, *LIN28*, and *NR5A2* (supplemental online Fig. 2A, 2B). For 3i/Lif medium‐derived clones, 72% (13 of 18) were free of transgenes (Fig. 2A), and 28% (5 of 18) contained residual transgenes (supplemental online Table 5). This suggests that using 3i/Lif medium is more advantageous for the generation of transgene‐free riPS cells (supplemental online Fig. 2C).

### Characterization of Transgene‐Free riPS Cells

Next, the obtained transgene‐free riPS cell lines were subjected to characterization by examining expression of pluripotency factors. In addition to the expression of ES cell typical surface marker of alkaline phosphatase (Fig. 2B), transgene‐free riPS cell lines also expressed multiple ES cell‐specific pluripotency markers (Fig. 2C). We also performed q‐PCR for a few selected pluripotency genes in transgene‐free riPS. The results further confirmed the successful activation of endogenous pluripotency factors in the transgene‐free riPS cells, but varied expression levels of these genes were noticed (supplemental online Fig. 3A).

To further characterize the pluripotency of our transgene‐free riPS lines, we next performed RNA‐seq analysis and compared the transcriptomes of rat embryonic fibroblasts (*n* = 3, independent cell lines), transgene‐free riPS cells (*n* = 3, independent clones), transgene‐intact riPS cells (*n* = 3, independent clones), and germline‐competent ES cells (*n* = 3, independent cell lines). Using principal component analysis to qualify the similarity between them, we found that transgene‐intact riPS cells are more similar to ES cells than transgene‐free riPS cells (supplemental online Fig. 3B). Heat map and hierarchical clustering also confirmed the similarity of transgene‐intact riPS cells and ES cells (Fig. 2D). Noticeably, removal of transgenes led to significant variations among transgene‐free riPS cells, which is consistent with our q‐PCR results previously mentioned. Next, we analyzed differentially expressed genes between transgene‐free riPS cells and rat ES cells (supplemental online Table 6). Although a few differentiation or developmental associated genes such as *Grhl3* and *Clu* were upregulated in transgene‐free iPS cells, no prominent pluripotency genes or stemness associated genes were found.

To further characterize the differentiation potential of transgene‐free riPS cells, the formation of embryoid bodies (EBs) in vitro and teratoma in vivo were conducted. The formed EBs could differentiate into cell types of ectoderm (nestin), mesoderm (α‐smooth muscle actin), and endoderm (GATA4) as validated by immunofluorescence staining (supplemental online Fig. 3C). Teratoma formation and subsequent tissue staining with H&E also demonstrate their multiple differentiation potential of forming epidermis structures (ectoderm), cartilage structures (mesoderm), and intestinal epithelium (endoderm) (supplemental online Fig. 3D). Collectively, these results indicate that our transgene‐free riPS cells display differentiation potential approximating that of ES cells.

**Figure 3:**
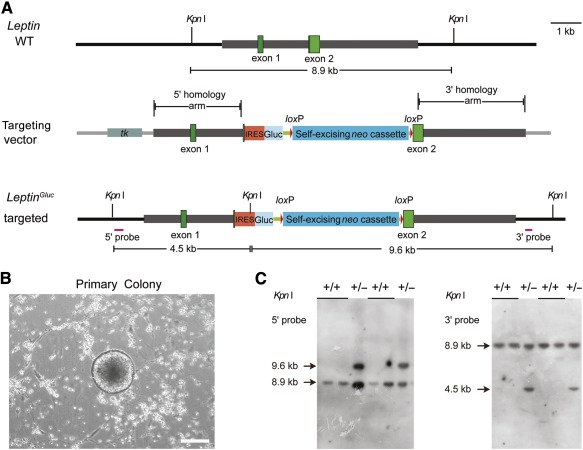
Gene targeting strategy for *Leptin* via homologous recombination and genotypic analysis of rat induced pluripotent stem (riPS) cells. **(A):** Schematic diagrams of wild‐type and gene targeted alleles of *Leptin*. The structure shows wild‐type rat *Leptin* allele and *Leptin^IRESGluc^* alleles that disrupt the *Leptin* gene via homologous recombination. The two exons of *Leptin* are shown as green boxes, the IRES sequence by orange box, the Gluc gene by cyan box, the self‐excising neo cassette by blue box, and *lox*P sites as red triangles. The 5′ and 3′ probes used to screen riPS cells are shown as purple lines. We obtained successfully targeted riPS cells through inserting the IRES, Gluc, and self‐excising neo cassette to disrupt the second exon of *Leptin*. If the desired recombination event happens, *Kpn* I digestion of genomic DNA from candidate rat ES cells or iPS cells will result in an 8.9 kb wild‐type band, 5′ and 3′ flanking probes are used to identify the additional downshifted band of 4.5 kb and upshifted band of 9.6 kb for the targeted allele, respectively. **(B):** Formation of typical, round, domed‐shaped colony that was picked and expanded for verification of gene targeting event. Scale bar = 200 μm. **(C):** Southern blot analysis of *Leptin* gene‐targeted riPS cells using 5′ and 3′ probes. DNA from riPS cells was digested with *Kpn* I. As shown in Figure 3A, the expected wild‐type band is 8.9 kb and *Leptin* gene‐targeted bands with 5′ and 3′ probes are 9.6 kb and 4.5 kb, respectively. Abbreviations: bla, ampicillin resistance gene; Gluc, Gaussia luciferase; IRES, Internal ribosome entry site; ori, origin of replication; *tk*, herpes simplex virus thymidine kinase gene; WT, wild‐type.

The ability of efficient gene targeting is a feature of high‐quality rat ES cells 63. We next designed a *Leptin* gene‐targeting vector based on homologous recombination to test whether transgene‐free riPS cell can mediate efficient gene targeting. In our targeting strategy, the *Leptin* gene was disrupted by inserting a *neo* selection cassette within exon 2 (Fig. 3A). G418 resistant clones were picked and expanded for further verification (Fig. 3B). By Southern blot analysis, 18.75% (24 of 128) of rat transgene‐free riPS clones were positive for the targeted mutation (Fig. 3C). In comparison, 16.6% (3 of 18) of ES cell clones were positive for the targeted mutation; therefore, no significant difference was observed between rat iPS and ES cells (supplemental online Table 7). From the point of suitability for gene targeting, the obtained transgene‐free riPS cells showed similar homologous recombination frequency to ES cells.

**Figure 4:**
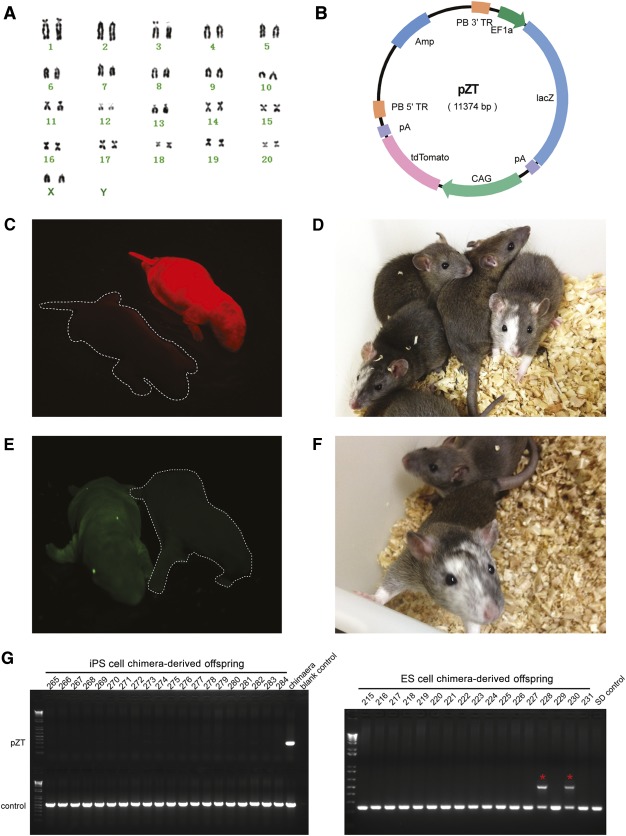
Generation of chimeras with riPS cells and ES cells. **(A):** Map of *piggyBac* based vector pZT. In the presence of *piggyBac* transposase, it can integrate into the genome, thus facilitating tracking of riPS cells derivatives. **(B):** Cytogenetic analysis (LSP‐24‐24, passage 12) indicated that the tdTomato marked transgene‐free rat induced pluripotent stem (riPS) cells had a normal female karyotype with 20 pairs of autosomal chromosomes and XX sex chromosomes. **(C):** Newborn pup exhibiting red florescence demonstrated the extensive contribution of riPS cells to the chimera. **(D):** Representative 1‐month‐old chimeras displayed typical hooded phenotype, signifying riPS cells took part in the development of chimera. We obtained 10 (5 males and 5 females) coat color chimeras for riPS cell line LSP‐24‐24. In addition, we obtained 11 coat color chimeras for riPS cell lines *Leptin*‐14 and *Leptin*‐18. **(E):** Newborn pup exhibiting green florescence demonstrated extensive contribution of rat ES cells to the chimera. **(F):** One‐month‐old chimera displayed typical hooded coat color phenotype, indicating that the ES cells took part in the development of chimera. We obtained 18 (10 males and 8 females) chimeras, and 3 male chimeras had germline transmission. **(G):** Polymerase chain reaction genotyping of chimera derived offspring. The chimeras were mated to wild‐type SD rats to produce offspring. No transgene was detected in the riPS cells‐derived offspring. In contrast, rat ES cell‐derived chimeras could routinely generate positive offspring (red asterisks) as confirmed by PCR genotyping. Abbreviations: ES, embryonic stem; iPS, induced pluripotent stem; SD, Sprague‐Dawley.

Karyotypic abnormalities have been a common problem during generation of rat ES and iPS cells 10, 11, 40, 64. To examine whether our optimized culture conditions helped maintain a stable karyotype, we performed metaphase chromosome analysis on riPS cell lines. We found that five tested clones maintained in 2i/Lif medium all had abnormal karyotypes, which exhibited as polyploid (>80% polyploid on average; supplemental online Fig. 3E). In contrast, four 3i/Lif‐derived clones all had normal karyotypes (>70% normal on average) even after long‐term culture (Fig. 4A, Table 1). This demonstrated that the inhibitor A83‐01 in 3i/Lif culture medium significantly improved chromosome stability.

**Table 1:** Summary of karyotype analysis of established rat iPS cell lines and rat ES cells

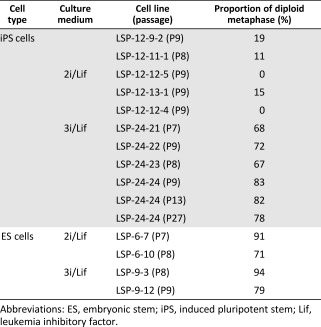

### Generation of Chimera Pups With riPS Cells and ES Cells

To further test the ability to contribute to the development of chimeric animals, transgene‐free riPS cells were injected into rat blastocysts, whereas ES cells were used as a control. To better track riPS cells, we first employed the *piggyBac*‐based vector named pZT to label riPS cell line LSP‐24‐24 with a red fluorescent protein (Fig. 4B). A total of 232 DA × Sprague‐Dawley hybrid recipient blastocysts 51 were injected with labeled riPS cells and transferred into eight day‐3.5 pseudopregnant Sprague‐Dawley females (Table 2). Of 40 live newborn pups, 10 were chimeric. The contribution of riPS cells to the development of chimeras was significant as evidenced by the strong detection of red fluorescence in chimeric animals (Fig. 4C). Chimeric animals exhibited typical hooded phenotype with coat color chimerism 65, indicating a high level contribution of riPS cells in chimeric animals (Fig. 4D). We next chose two correctly *Leptin*‐targeted riPS cell lines for blastocyst injection experiments (Table 2), and 11 chimeras were obtained with typical coat color chimerism. In the control injection experiment with Sprague‐Dawley ES cells labeled with a green fluorescent protein transgene, we obtained 64 newborn pups and 18 were chimeric indicated by green fluorescence and coat color chimerism (Fig. 4E, 4F). Details for chimera generation are displayed in Table 2. riPS cells showed comparable ability in chimeric contribution.

**Table 2:** Rat chimera generation from iPS cells and ES cells

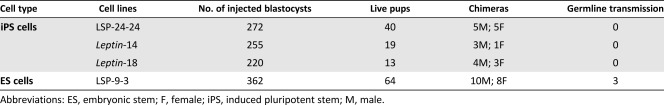

For the 3 riPS cell lines‐derived chimeric animals, all of the 21 chimeras (12 males and 9 females) were bred to Sprague‐Dawley mates for more than 12 months. More than 1100 pups were produced but none of them showed germline transmission. PCR genotyping of all these pups did not detect any transgene (partial genotyping results are shown in Fig. 4G). In contrast, nine ES cell‐derived male chimeric animals were used for breeding to Sprague‐Dawley mates and three of them produced offspring with verified germline transmission (Fig. 4G).

## Discussion

To date, high‐quality transgene‐free iPS cells have been reported only in mice. In the current study, we used episomal vectors to obtain transgene‐free iPS cells in rat. Despite not obtaining germline transmission, these cells exhibit typical characteristics of pluripotent stem cells and contribute significantly to chimeric formation. Although these results are encouraging, additional work is required to derive authentic ground‐state riPS cells, a required step before we can realize large animal iPS cells. Several lessons can be learned from reprogramming of transgene‐free riPS cells that may provide valuable clues for overcoming the difficulties of acquiring ground‐state iPS/ES cells in other animals.

First, we found reduced oxygen culture conditions significantly promoted the reprogramming process. Furthermore, the majority (62.5%–77.1%) of screened primary clones could be converted to stable cell lines (supplemental online Table 4), which is a dramatic improvement compared with the previously reported 20%–30% obtained for riPS cell establishment 40, 43. Although hypoxia plays a crucial role in the reprogramming process of somatic cells, we found it dispensable for the propagation of riPS cells. Established cell lines could be maintained in normoxic conditions for up to 40 passages without differentiation and still maintained the potential for extensive contribution to chimeric formation.

Second, we determined that the *miR‐302/367* gene cluster plays an important role in reprogramming rat embryonic fibroblasts into iPS cells. Previous reports have also found that the *miR‐302/367* gene cluster increased reprogramming in mice and human 66, 67, 68. In our initial study using pMaster3, which contains no *miR‐302/367* gene cluster, we failed to generate riPS cells under normoxia culture conditions. Although we obtained riPS cells with pMaster3 under hypoxia culture conditions, the efficiency was suboptimal. By including the human or rat‐specific *miR‐302/367* gene cluster in the reprogramming gene cocktail, the reprogramming efficiencies were greatly increased. Moreover, compared with the human *miR‐302/367* gene cluster, the rat‐specific *miR‐302/367* gene cluster was more efficient. In the future, *miR‐302/367* combined with other favorable factors should promote the realization of complete reprogramming of iPS cells in other species.

Third, we found that 3i medium contributed to the stability of riPS cell karyotypes. Because the 2i medium was successfully used to generate the first rat ES cell lines, ES cells from other rat strains have also been established 51, 52, 69, 70. However, how to maintain stable karyotype of riPS cells and ES cells is still an unresolved issue 10, 11, 40, which impedes more broad scientific applications. Previous studies showed that TGF‐β inhibitor A83‐01 together with MEK inhibitor PD0325901, and GSK3β inhibitor CHIR99021, result in significant increases in reprogramming efficiency 71, 72. Although 3i/Lif medium did not significantly improve reprogramming efficiency in our hands, it significantly increased the stability of karyotypes of either riPS cells or ES cells, but especially riPS cells (Table 1), although the underlying mechanism is still unclear.

Fourth, endogenous gene expression levels of riPS cells vary from line to line, and this may have affected the ability for germline transmission. Our transgene‐free riPS cells could be successfully used for gene targeting and generated high‐percentage chimeras, but they were not able to give rise to germline transmission. One of the reasons may be the variation of endogenous gene expression in riPS cells compared with ES cells. Previous reports indicated that iPS cell gene expression signatures are different from those for ES cells 73, 74. After transgene removal, both q‐PCR and RNA‐seq analysis results indicate the expression levels of pluripotency genes vary among the three transgene‐free riPS cell lines tested. These suggest that technology for generating transgene‐free riPS cells still needs further optimization, including finding novel reprogramming factors or better culture conditions. Therefore, in the future, establishing iPS cells with a homogeneous gene expression profile similar to ES cells may be essential to obtain germline competency.

Finally, the inner cell mass‐derived mouse ES cells represent a naïve phase of pluripotency; they meet all criteria of pluripotency including capability for chimera formation, germline transmission 12, 75, 76, and generation of entirely ES cell‐derived animals through tetraploid complementation 77. The somatic cell‐derived mouse iPS cells can achieve the same developmental properties as their counterpart ES cells 15, 78. In contrast, the postimplantation embryos‐derived EpiSCs 79, 80 exist in a distinct state named primed pluripotent state 76. These EpiSCs exhibit some pluripotency feature but have limited developmental potential, and in particular they rarely contribute to chimera formation.

For rats, the primed rat ES cells showed poor ability of chimerism contribution 79. The germline‐competent rat ES cells have been derived from several strains such as Sprague‐Dawley 11, 51, DA 10, 11, Wistar 52, Long‐Evans agouti 52 and Fischer 344 70; however, no one has been able to produce all ES cell‐derived animals using these cells in tetraploid complementation, suggesting that more efforts are needed to bring rat ES cells to the authentic naïve state. In comparison, riPS cells reported to date are far from ideal. In the limited reports of riPS cell generation, germline‐competent riPS cells have only been achieved in Wistar rats and DA rats 39, 41. However, these cells were generated with lentivirus and retained virus integration. Therefore, the significant challenge remains for the riPS field (as well as for large animals including humans). That is, can transgene‐free riPS cells be generated that have capabilities for chimeric formation and germline transmission?

## Conclusion

We have successfully derived transgene‐free riPS cells, which contributed to chimeric formation, although no germline transmission has been obtained to date. This in vivo study will provide a basis for future work to isolate germline‐competent riPS cells and iPS cells for other animal species.

## Author Contributions

S.L. and H.L.: conception and design, collection and/or assembly of data, data analysis and interpretation, manuscript writing; H.M. and Y.W.: data collection, data analysis and interpretation; N.L., M.R.C., and E.C.B.: advice and supervision; S.W.: conception and design, financial support, data analysis and interpretation, manuscript writing, final approval of manuscript.

## Disclosure of Potential Conflicts of Interest

The authors indicated no potential conflicts of interest.

## Supporting information

Supporting Information
