# Supplementary material for: Derivation of Transgene‐Free Rat Induced Pluripotent Stem Cells Approximating the Quality of Embryonic Stem Cells
Source: Stem Cells Transl Med. 2016 Sep 13;6(2):340–51. doi: 10.5966/sctm.2015-0390 (PMC5442795; doi:10.5966/sctm.2015-0390)
Supplement: Supplementary file 1 — Supporting Information [file SCT3-6-340-s001.pdf]

Supporting Information Table S1 Rat embryonic stem cell derivation efficiency

Culture medium	No. of embryos	Established lines	Efficiency (%)
2i medium	10	2	20
3i medium	25	15	60

Supporting Information Table S2 List of reprogramming vectors used in this study

Vectors	Combination of transcription factors
pMaster3	*hOCT4*，*hSOX2*，*hKLF4*，*hC-MYC*，*hNANOG*，*hLIN28*，*hNR5A2*
pMaster12	*hOCT4*，*hSOX2*，*hKLF4*，*hC-MYC*，*hNANOG*，*hLIN28*，*hNR5A2*，*hMIR302/367*
pMaster22	*hOCT4*，*hSOX2*，*hKLF4*，*hC-MYC*，*hNANOG*，*hLIN28*，*hNR5A2*，*rat mir302/367*

Supporting Information Table S3 Reprogramming efficiencies of each vector

Vector	Culture condition	Reprogramming efficiency
pMaster3	Normoxia	0
pMaster3	Hypoxia (5% O_2)	~0.0004%
pMaster12	Normoxia	~0.001%
pMaster12	Hypoxia (5% O_2)	~0.01%
pMaster22	Normoxia	~0.002%
pMaster22	Hypoxia (5% O_2)	~0.02%

Supporting Information Table S4 Rat iPS cells establishment efficiency

Experiment No.	Induction vector	Passage of REFs*	Culture medium	Picked colonies	Established lines	Culture condition	Establishment Efficiency (%)
LSP-12	pMaster12	P5**	2i/Lif	25	15	Hypoxia	52
LSP-13	pMaster22	P5	2i/Lif	32	13	Hypoxia	41
LSP-17	pMaster12	P1	2i/Lif	8	6	Hypoxia	75
LSP-18	pMaster22	P1	2i/Lif	8	5	Hypoxia	62.5
LSP-19	pMaster3	P1	2i/Lif	8	4	Hypoxia	50
LSP-20	pMaster12	P1	2i/Lif	0	0	Normoxia	0
LSP-22	pMaster12	P1	3i/Lif	12	2	Normoxia	17
LSP-23	pMaster22	P1	3i/Lif	12	2	Normoxia	17
LSP-24	pMaster12	P1	3i/Lif	48	37	Hypoxia	77.1
LSP-28	pMaster22	P1	3i/Lif	8	5	Hypoxia	62.5

*REFs = rat embryonic fibroblasts

**P5 means passage 5

Supporting Information Table S5 PCR analysis results of rat iPS cells

Cell line	Culture medium	Reprogramming vector	Culture condition	Transgene-free
LSP-12-10-1	2i	pMaster12	Hypoxia (5% O2)	N
LSP-12-10-2	2i	pMaster12	Hypoxia (5% O2)	N
LSP-12-11-1	2i	pMaster12	Hypoxia (5% O2)	Y
LSP-12-11-3	2i	pMaster12	Hypoxia (5% O2)	Y
LSP-12-11-5	2i	pMaster12	Hypoxia (5% O2)	Y
LSP-12-11-6	2i	pMaster12	Hypoxia (5% O2)	N
LSP-12-12-1	2i	pMaster12	Hypoxia (5% O2)	Y
LSP-12-12-2	2i	pMaster12	Hypoxia (5% O2)	N
LSP-12-12-4	2i	pMaster12	Hypoxia (5% O2)	Y
LSP-12-12-5	2i	pMaster12	Hypoxia (5% O2)	Y
LSP-12-12-6	2i	pMaster12	Hypoxia (5% O2)	Y
LSP-12-1-3	2i	pMaster12	Hypoxia (5% O2)	N
LSP-12-2-4	2i	pMaster12	Hypoxia (5% O2)	N
LSP-12-2-5	2i	pMaster12	Hypoxia (5% O2)	N
LSP-12-2-6	2i	pMaster12	Hypoxia (5% O2)	Y
LSP-12-4-1	2i	pMaster12	Hypoxia (5% O2)	N
LSP-12-4-2	2i	pMaster12	Hypoxia (5% O2)	N
LSP-12-4-3	2i	pMaster12	Hypoxia (5% O2)	N
LSP-12-5-1	2i	pMaster12	Hypoxia (5% O2)	Y

LSP-12-5-2	2i	pMaster12	Hypoxia (5% O2)	N
LSP-12-5-3	2i	pMaster12	Hypoxia (5% O2)	Y
LSP-12-5-4	2i	pMaster12	Hypoxia (5% O2)	N
LSP-12-5-5	2i	pMaster12	Hypoxia (5% O2)	Y
LSP-12-6-1	2i	pMaster12	Hypoxia (5% O2)	N
LSP-12-9-2	2i	pMaster12	Hypoxia (5% O2)	Y
LSP-12-9-4	2i	pMaster12	Hypoxia (5% O2)	N
LSP-12-9-6	2i	pMaster12	Hypoxia (5% O2)	Y
LSP-13-13-4	2i	pMaster22	Hypoxia (5% O2)	N
LSP-13-13-5	2i	pMaster22	Hypoxia (5% O2)	Y
LSP-13-14-1	2i	pMaster22	Hypoxia (5% O2)	Y
LSP-13-14-3	2i	pMaster22	Hypoxia (5% O2)	N
LSP-13-16-2	2i	pMaster22	Hypoxia (5% O2)	N
LSP-13-16-3	2i	pMaster22	Hypoxia (5% O2)	N
LSP-13-7-1	2i	pMaster22	Hypoxia (5% O2)	N
LSP-13-9-5	2i	pMaster22	Hypoxia (5% O2)	Y
LSP-13-9-6	2i	pMaster22	Hypoxia (5% O2)	Y
LSP-24-20-1	3i	pMaster12	Hypoxia (5% O2)	Y
LSP-24-12	3i	pMaster12	Hypoxia (5% O2)	N
LSP-24-20	3i	pMaster12	Hypoxia (5% O2)	Y
LSP-24-19	3i	pMaster12	Hypoxia (5% O2)	N
LSP-24-21	3i	pMaster12	Hypoxia (5% O2)	Y

LSP-24-22	3i	pMaster12	Hypoxia (5% O2)	Y
LSP-24-23	3i	pMaster12	Hypoxia (5% O2)	Y
LSP-24-23-3	3i	pMaster12	Hypoxia (5% O2)	Y
LSP-24-20-3	3i	pMaster12	Hypoxia (5% O2)	Y
LSP-24-24	3i	pMaster12	Hypoxia (5% O2)	Y
LSP-24-3	3i	pMaster12	Hypoxia (5% O2)	N
LSP-25-5-2	3i	pMaster12	Hypoxia (5% O2)	Y
LSP-27-2-1	3i	pMaster12	Hypoxia (5% O2)	Y
LSP-26-2-1	3i	pMaster22	Hypoxia (5% O2)	N
LSP-26-3-4	3i	pMaster22	Hypoxia (5% O2)	Y
LSP-26-4-2	3i	pMaster22	Hypoxia (5% O2)	N
LSP-26-6-2	3i	pMaster22	Hypoxia (5% O2)	Y
LSP-28-1-2	3i	pMaster22	Hypoxia (5% O2)	Y

Y=Yes; N=No

Supporting Information Table S6 List of differentially exppressed genes in transgene-free rat iPS cells vs ES cells

	Gene_symbol	Base Mean	$\log_2$(Fold Change)	lfcSE	stat	*p* Value	padj
ENSRNOG00000047706	RT1-CE16	34.5394	-6.1057	1.3237	-4.6127	3.97E-06	0.0038
ENSRNOG00000060849	Rcor2	17.8088	-5.2608	1.0745	-4.8959	9.79E-07	0.0014
ENSRNOG00000007523	Cct6b	46.8523	-5.1359	1.0149	-5.0604	4.18E-07	0.0010
ENSRNOG00000015401	Mapk4	159.5972	-2.1030	0.4663	-4.5100	6.48E-06	0.0050
ENSRNOG00000003745	Atf3	116.1880	-1.9472	0.4842	-4.0214	5.78E-05	0.0206
ENSRNOG00000020202	Asrgl1	47.3198	-1.7402	0.3737	-4.6568	3.21E-06	0.0034
ENSRNOG00000048861	LOC498122	122.4874	-1.5498	0.2792	-5.5515	2.83E-08	9.11E-05
ENSRNOG00000019802	Zfp428	531.3881	-1.3415	0.3259	-4.1158	3.86E-05	0.0174
ENSRNOG00000004430	Cep131	245.8407	-1.1508	0.2937	-3.9179	8.93E-05	0.0248
ENSRNOG00000001517	Pdk1	430.8831	-1.0696	0.2203	-4.8550	1.20E-06	0.0016
ENSRNOG00000002373	Akap1	966.6341	1.0321	0.1724	5.9856	2.16E-09	1.16E-05
ENSRNOG00000029971	ND5	11490.0973	1.1299	0.2902	3.8938	9.87E-05	0.0269
ENSRNOG00000021056	Kcnj14	88.0326	1.3264	0.3033	4.3733	1.22E-05	0.0070
ENSRNOG00000011879	Nfat5	741.9896	1.3586	0.3174	4.2808	1.86E-05	0.0103
ENSRNOG00000019573	Lcat	42.5623	1.3929	0.3363	4.1415	3.45E-05	0.0163
ENSRNOG00000030644	ND1	16387.5236	1.4310	0.2979	4.8029	1.56E-06	0.0019
ENSRNOG00000024066	Fundc2	134.4565	1.5605	0.3440	4.5366	5.72E-06	0.0046
ENSRNOG00000001271	Card6	139.2871	1.8049	0.3110	5.8033	6.50E-09	2.62E-05

ENSRNOG00000020585	Tbxa2r	51.6061	2.3676	0.5643	4.1957	2.72E-05	0.0137
ENSRNOG00000029427	Grhl3	120.2871	2.4612	0.6266	3.9281	8.56E-05	0.0242
ENSRNOG00000014427	Chrnb4	25.6065	2.6140	0.5870	4.4533	8.46E-06	0.0061
ENSRNOG00000006653	Slc38a4	602.4645	2.7891	0.4284	6.5112	7.46E-11	6.00E-07
ENSRNOG00000014761	Rasd2	23.9922	2.7897	0.7194	3.8779	0.0001	0.0283
ENSRNOG00000013027	Rgl3	25.6125	2.8101	0.7434	3.7799	0.0002	0.0377
ENSRNOG00000009620	Cybrd1	112.9714	2.9425	0.7267	4.0492	5.14E-05	0.0197
ENSRNOG00000005277	Ptprv	213.7536	2.9958	0.6445	4.6480	3.35E-06	0.0034
ENSRNOG00000013835	Timm8a2	15.0403	2.9987	0.7626	3.9323	8.41E-05	0.0242
ENSRNOG00000032231	Mroh6	10.4090	3.1344	0.8285	3.7831	0.0002	0.0377
ENSRNOG00000014683	Il1rl2	202.0463	3.1362	0.7148	4.3877	1.15E-05	0.0068
ENSRNOG00000014504	Il1r1	279.0232	3.2094	0.7554	4.2484	2.15E-05	0.0115
ENSRNOG00000001414	Serpine1	2721.5855	3.5571	0.8854	4.0175	5.88E-05	0.0206
ENSRNOG00000001195	Trpv4	12.5600	3.5756	0.9582	3.7314	0.0002	0.0436
ENSRNOG00000054438	LOC292543	109.6941	3.6031	0.4471	8.0593	7.68E-16	1.24E-11
ENSRNOG00000005572	RGD1306782	69.9785	3.6059	0.7254	4.9706	6.68E-07	0.0011
ENSRNOG00000013291	Cyp2c23	7.3644	3.7179	0.9971	3.7288	0.0002	0.0436
ENSRNOG00000014361	Edn1	69.4878	3.8871	1.0346	3.7570	0.0002	0.0401
ENSRNOG00000047367	Card14	294.8559	3.9451	0.9889	3.9894	6.62E-05	0.0219
ENSRNOG00000009118	Pou2f3	6.6205	3.9772	0.9676	4.1106	3.95E-05	0.0174
ENSRNOG00000016460	Clu	1171.7083	3.9810	0.9384	4.2421	2.21E-05	0.0115
ENSRNOG00000012347	Gata2	51.4198	3.9997	0.9588	4.1715	3.03E-05	0.0148

ENSRNOG00000011800	F3	495.6745	4.0093	0.9916	4.0432	5.27E-05	0.0197
ENSRNOG00000028531	Ccl25	27.1723	4.0680	0.8204	4.9583	7.11E-07	0.0011
ENSRNOG00000021242	Adam33	122.4006	4.2129	1.0578	3.9826	6.82E-05	0.0219
ENSRNOG00000051548	Lmod1	290.7254	4.2408	1.0737	3.9496	7.83E-05	0.0233
ENSRNOG00000008697	Nov	469.7524	4.2533	1.0785	3.9438	8.02E-05	0.0235
ENSRNOG00000032240	Gbp5	10.8610	4.3675	0.9855	4.4316	9.36E-06	0.0061
ENSRNOG00000050040	Akr1c12	23.2951	4.3796	1.1009	3.9782	6.94E-05	0.0219
ENSRNOG00000033706	Klk14	12.5645	4.4543	1.0988	4.0538	5.04E-05	0.0197
ENSRNOG00000004400	Avpr1a	176.1344	4.4982	1.1374	3.9547	7.66E-05	0.0233
ENSRNOG00000019757	Dpep3	7.3221	4.9564	1.1157	4.4423	8.90E-06	0.0061
ENSRNOG00000021345	Timd2	9.3230	4.9851	1.2561	3.9686	7.23E-05	0.0224
ENSRNOG00000012789	Trhr2	29.8242	5.0345	1.0820	4.6529	3.27E-06	0.0034
ENSRNOG00000019445	Msln	24.2446	5.7523	1.3015	4.4197	9.88E-06	0.0061

Down-regulated in ES cells

Up-regulated in ES cells

Supporting Information Table S7. ***Leptin*** **gene targeting frequency in transgene-free rat iPS cells and ES cells**

	Cell line	Target genes	No. of candidate colonies	No. of targeted colonies	Targeting frequency
**iPS cells**	LSP-24-24	*Leptin*	128	24	18.8%
**ES cells**	LSP-9-3	*Leptin*	18	3	16.6%

Supporting Information Table S8. Primers used in this study

PCR amplified region	Primer Sequence (5'-3')	Production length (bp)
for q-PCR		
Oct4	F: AGCATACGAGTTCTGTGGAGGGA	352
	R: GATGGTTGTCTGGCTGAACACCT	
Rex1	F: AAAAGGTGGCATATGACCGCAGT	230
	R: AGTCCCTTTCAGCTCCTCTACCC	
Klf4	F: GGACCCAGTATACATTCCGCCAC	369
	R: AGTTCCTCGGGACTCAGTGTAGG	
Sox2	F: GGAGAACCCCAAGATGCACAACT	205
	R: TCCGGGAAGCGTGTACTTATCCT	
Eras	F:TCTTTGCTCTTGATGACCCGTCG	220
	R: AAAGCTTCCTCTACACCTTGCCG	
Nanog	F: CCTACCTCTTCAAGATAGCCCTG	184
	R: CCTTTGCCTCTGAAACCTATCCT	
Gapdh	F: TTGCCATCAACGACCCCTTCATT	211
	R: ACGCCAGTAGACTCCACGACATA	
for PCR		
neo-IRES 1	WS1384: ATGATGGATACTTTCTCGGCAGGA	588
	WS1385: TGCCACGTTGTGAGTTGGATAGTT	

neo-IRES 2	WS1386:GGACAGGTCGGTCTTGACAAAAAG	569
	WS1387: TCTGTTGAATGTCGTGAAGGAAGC	
tk 1	WS1388: AATCCAGGATAAAGACGTGCATGG	701
	WS1389: GACAATCGCGAACATCTACACCAC	
tk 2	WS1390: ATACCGCACCGTATTGGCAAGTAG	506
	WS1391: ACGTACCCGAGCCGATGACTTACT	
MYC-*SOX2* 1	WS1392: GAAGTTCTCCTCCTCGTCGCAGTA	502
	WS1393: CCTGCAGTACAACTCCATGACCAG	
MYC-*SOX2* 2	WS1394: GTCGCAGATGAAACTCTGGTTCAC	616
	WS1395: TGTGGTTACCTCTTCCTCCCACTC	
SOX2-*KLF4* 1	WS1396: TTCTCCGTCTCCGACAAAAGTTTC	536
	WS1397: AAGAGTTCCCATCTCAAGGCACAC	
SOX2-*KLF4* 2	WS1398: CCTTCTTCATGAGCGTCTTGGTTT	545
	WS1399: TGAACTGACCAGGCACTACCGTAA	
KLF4-*OCT4* 1	WS1400: GATCGTTGAACTCCTCGGTCTCTC	618
	WS1401: ATGTGGTCCGAGTGTGGTTCTGTA	
KLF4-*OCT4* 2	WS1402: GGGTCAGCGAATTGGAGAGAATAA	612
	WS1403: GATCAAGCAGCGACTATGCACAAC	
oriP 1	WS1404: ATGGCTATGGGCAACACATAATCC	523
	WS1405: CTCTCAGCGACCTCGTGAATATGA	
oriP 2	WS1406: CACAAACCCCTTGGGCAATAAATA	500
	WS1407: CCATTAGTGGTTTTGTGGGCAAGT	

EBNA 1	WS1408: CCTCATCTCCATCACCTCCTTCAT	487
	WS1409: TCCAACCCGAAATTTGAGAACATT	
EBNA 2	WS1410:GGAAACCAGGGAGGCAAATCTACT	357
	WS1411:TCACGTAGAAAGGACTACCGACGA	
NANOG-LIN28 1	WS1416: AACCCTTCCATGTGCAGCTTACTC	436
	WS1417: CTGCTGGGGAAGGCCTTAATGTA	
NANOG-LIN28 2	WS1418: CGCCTCTCACTCCCAATACAGAAT	442
	WS1419: GAAGTGGCGTGAAACAGACTTTGA	
NR5A2 1	WS1555: TGTCAATTTGGCAGTTCTGGTTTT	449
	WS1556: AGGGGTTTTATGCGATGGAGTTTC	
NR5A2 2	WS1557: GGACAACGCTTTCTCTGTGTTTTG	415
	WS1558: CCAGCTTGGCACTTGATGTAATTC	
EF1a-*OCT4* 1	WS1551: GCACTAGCCCCACTCCAACCT	445
	WS1552: AGGGGTTTTATGCGATGGAGTTTC	
EF1a-*OCT4* 2	WS1553: GAGTTGCTCTCCACCCCGACT	453
	WS1554: CCAGCTTGGCACTTGATGTAATTC	
miR 302/367	WS1645:TCTCCTACTTATTTCTCACCCCGATG	317
	WS1646: GGAAATCATGATCATCCCTTCTCCT	
pZT	F:GGATCTGTAGGGCGCAGTAG	317
	R:AGACCCCTAGGAATGCTCGT	

F: Forward;
R: Reverse

Figure S1

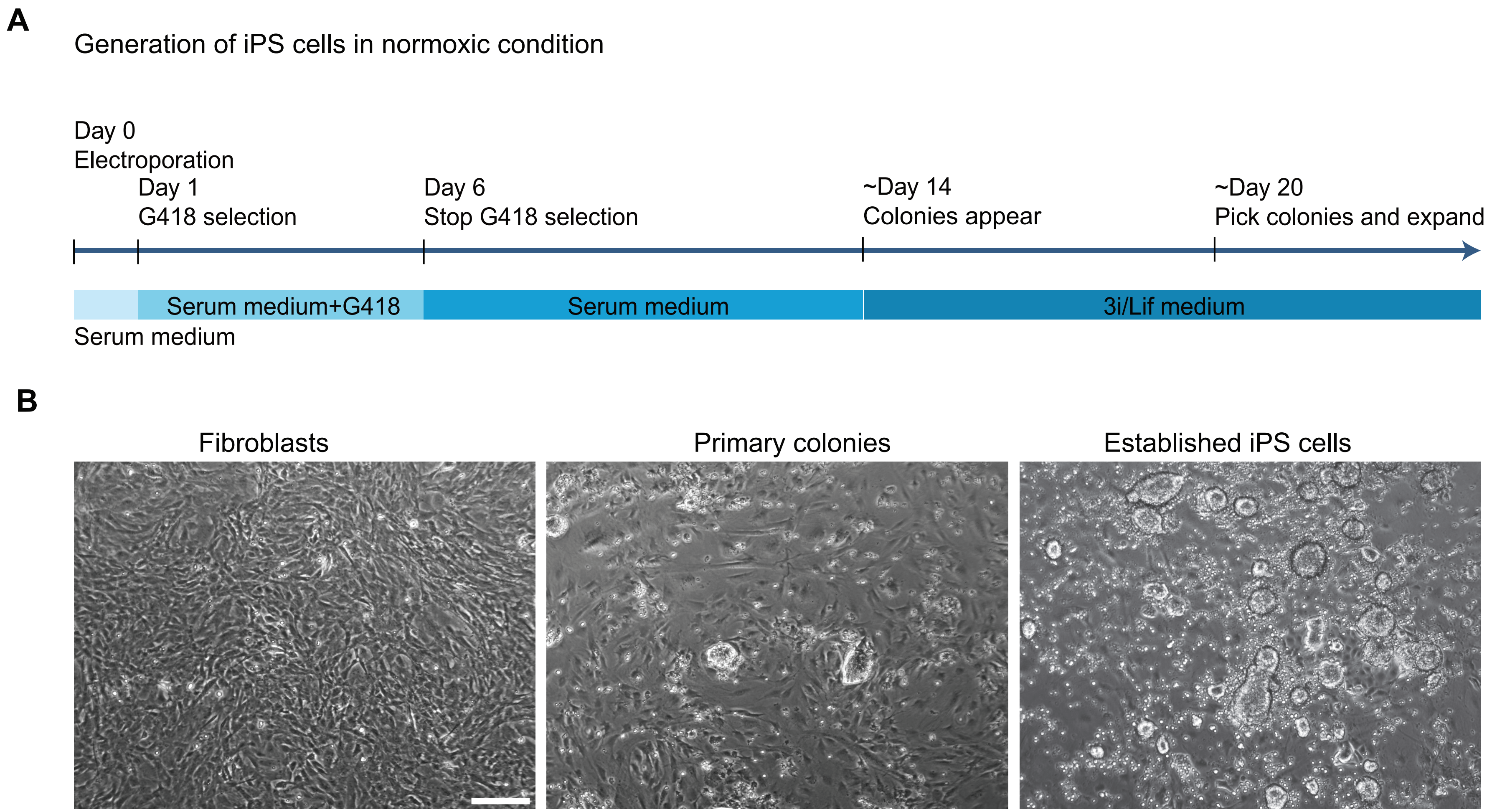
A
Generation of iPS cells in normoxic condition
Day 0
Electroporation
Day 1
G418 selection
Day 6
Stop G418 selection
~Day 14
Colonies appear
~Day 20
Pick colonies and expand
Serum medium+G418
Serum medium
3i/Lif medium
Serum medium
B
Fibroblasts
Primary colonies
Established iPS cells

Figure S2

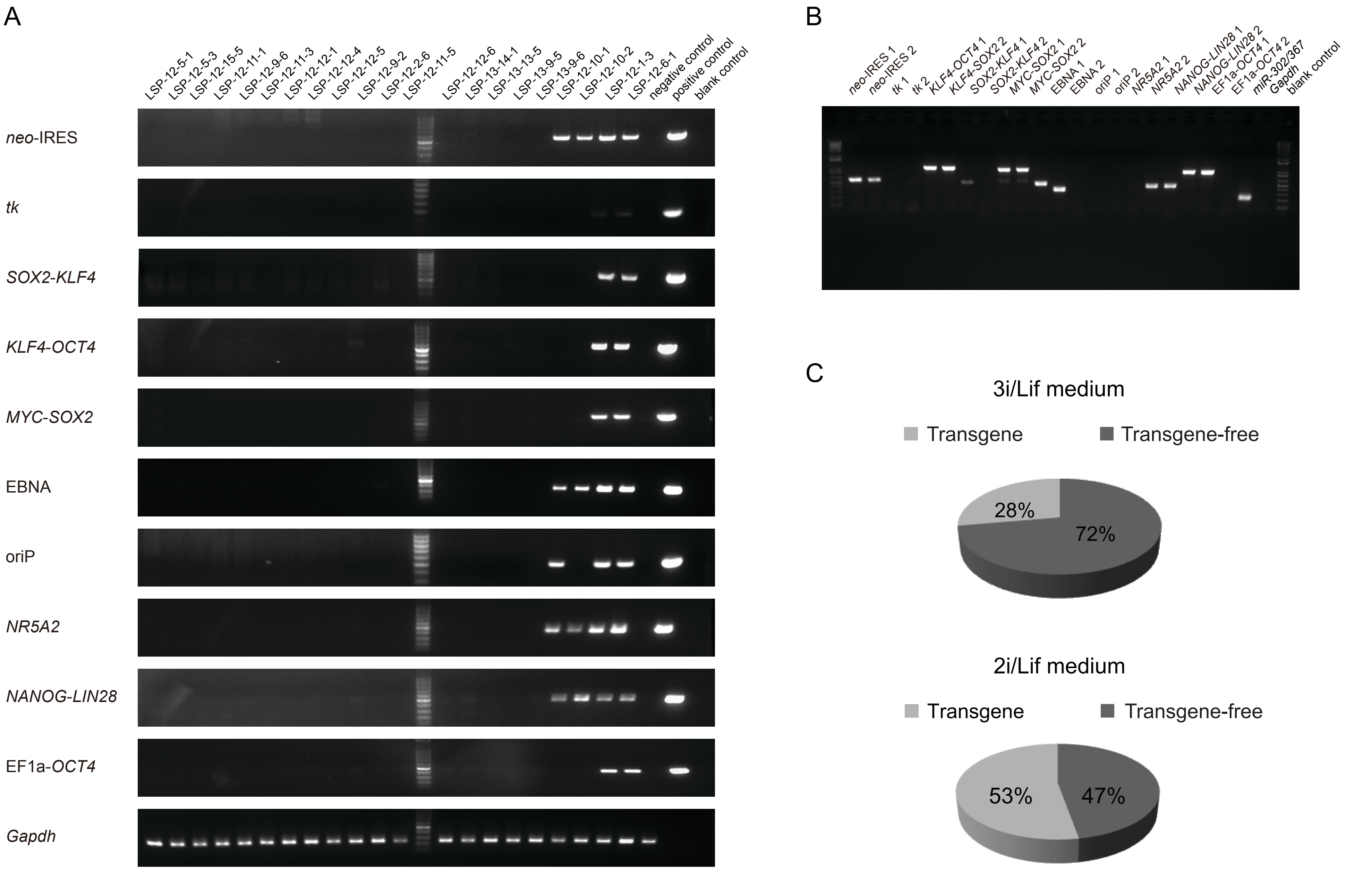
A
LSP-12-5-1
LSP-12-5-3
LSP-12-15-5
LSP-12-11-1
LSP-12-9-6
LSP-12-11-3
LSP-12-12-1
LSP-12-12-4
LSP-12-12-5
LSP-12-9-2
LSP-12-2-6
LSP-12-11-5
LSP-12-12-6
LSP-13-14-1
LSP-13-13-5
LSP-13-9-5
LSP-13-9-6
LSP-12-10-1
LSP-12-10-2
LSP-12-1-3
LSP-12-6-1
negative control
positive control
blank control
neo-IRES
tk
SOX2-KLF4
KLF4-OCT4
MYC-SOX2
EBNA
oriP
NR5A2
NANOG-LIN28
EF1a-OCT4
Gapdh
B
neo-IRES 1
neo-IRES 2
tk 1
tk 2
KLF4-OCT4 1
KLF4-SOX2 2
SOX2-KLF4 1
SOX2-KLF4 2
MYC-SOX2 1
MYC-SOX2 2
EBNA 1
EBNA 2
oriP 1
oriP 2
NR5A2 1
NR5A2 2
NANOG-LIN28 1
NANOG-LIN28 2
EF1a-OCT4 1
EF1a-OCT4 2
miR-302/367
Gapdh
blank control
C
3i/Lif medium
Transgene
Transgene-free
28%
72%
2i/Lif medium
Transgene
Transgene-free
53%
47%

Figure S3

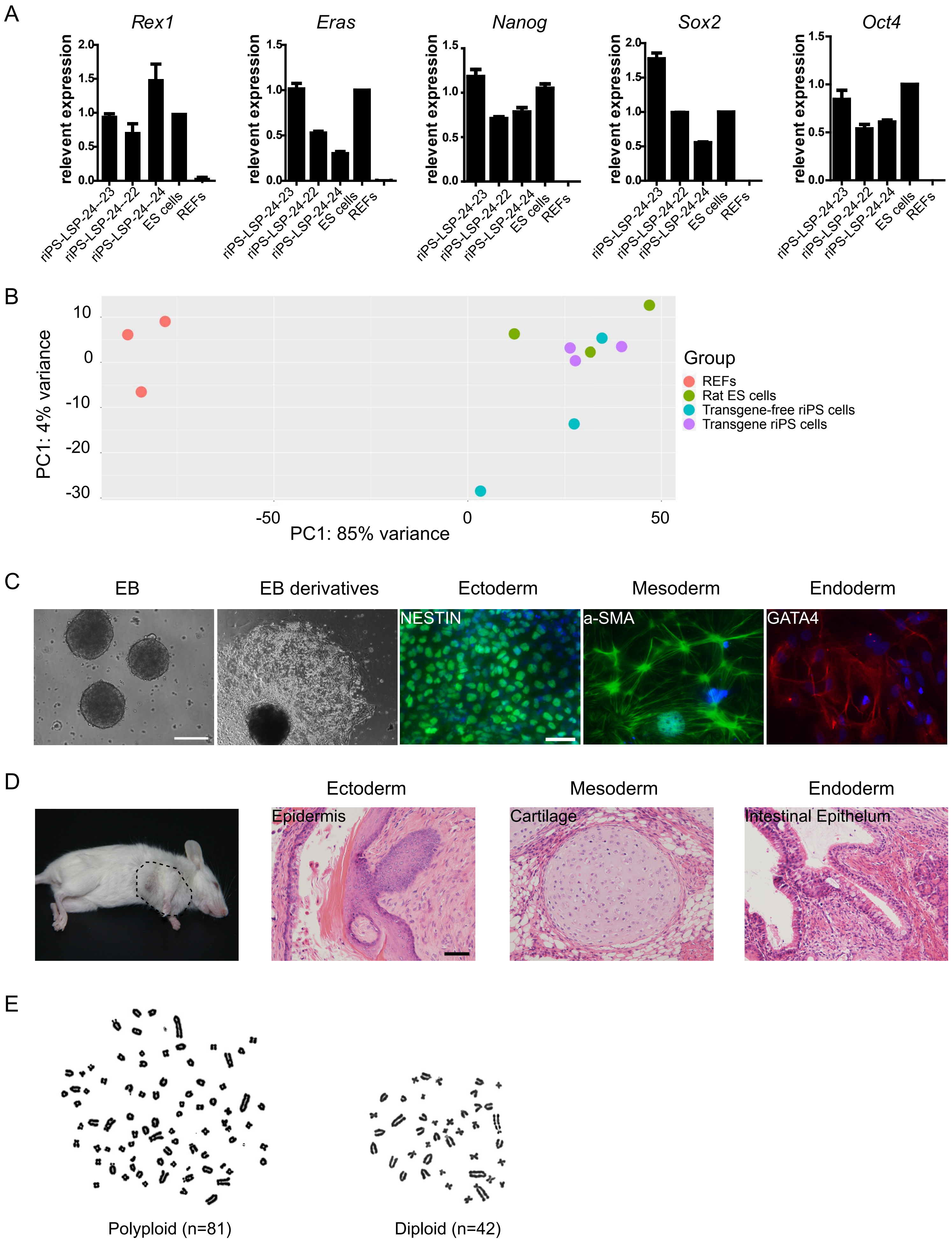
A
Rex1
Eras
Nanog
Sox2
Oct4
relevent expression
riPS-LSP-24-23
riPS-LSP-24-22
riPS-LSP-24-24
ES cells
REFs
B
PC1: 4% variance
PC1: 85% variance
Group
REFs
Rat ES cells
Transgene-free riPS cells
Transgene riPS cells
C
EB
EB derivatives
Ectoderm
Mesoderm
Endoderm
NESTIN
a-SMA
GATA4
D
Ectoderm
Mesoderm
Endoderm
Epidermis
Cartilage
Intestinal Epithelum
E
Polyploid (n=81)
Diploid (n=42)

Supplemental Figure Legends

Figure S1. Reprogramming of REFs in normal condition. **(A):** Flow chart of iPS generation in normal condition. **(B):** Representative example of riPS cells in normal condition. The primary colonies tightly adhered to the culture dish and after subsequent passage, formed riPS cell without clear boundary to the feeder cells. Scale bar = 200 um.

Figure S2. PCR verification of transgene-free riPS cells. **(A):** PCR analysis of candidate riPS cells. Twenty-one riPS cell lines were analyzed by PCR with 10 pairs of primers covering the reprogramming vectors pMaster12 and pMaster22. Seventeen of them are completely transgene-free and the remaining 4 colonies still have residual exogenous gene sequences. Rat embryonic fibroblast (REF) genomic DNA was used as a negative control. Genomic DNA of nuclear transfected REFs was used as positive control. PCR without genomic template was used as blank control. **(B):** PCR analysis of riPS cell line LSP-24-3. We performed PCR with 21 pairs of primers to detect vector fragments in the riPS cell line. The drug selection marker *neo* and transcription factors were retained. PCR without genomic template was used as blank control. **(C):** Frequency of transgene removal in clones obtained with or without A83-01. Addition of A83-01 in 2i/Lif medium promoted the frequency of transgene-free riPS cells improved from 47% to 72%.

Figure S3. Additional characterization of riPS cells *in vitro* and *in vivo*. **(A):** The expression levels of endogenous pluripotency factors quantified by q-PCR. We chose 3 different transgene-free riPS cell lines for q-PCR test. The graphs showed that the endogenous *Rex1*,

Eras, *Nanog*, *Sox2* and *Oct4* of established riPS cells were activated, in comparison with rat ES cells, though with variation across different clones. Gene expression levels were normalized to expression of the house-keeping gene glyceraldehyde-3-phosphate dehydrogenase (*Gapdh*). The RNA expression of pluripotency factors in rat ES cells was set to 1, rat embryonic fibroblasts were used as a negative control. Data indicates the means ± SD of 3 independent technical repeats. **(B):** Principal component analysis of the transcriptomes of REFs, transgene-free riPS cells (LSP-24-22, LSP-24-23, LSP-24-24), transgene-intact riPS cells (LSP-24-11, LSP-24-12, LSP-24-19) and rat ES cells (SD-Tg.EC1, DAc2, DAc8) [11, 51]. **(C):** Embryoid bodies (EBs) derived from riPS cells and subsequent differentiated derivatives. We obtained EB formation using riPS cells (LSP-24-24, passage 25) by continuous culture in differentiation medium, which was changed every two days sequentially with serum medium supplemented with 50%, 30% and 0% 3i/Lif medium. Additional culture in serum medium for another week resulted in different cell types. Scale bar, 500 μm. Immunofluorescence staining confirmed *in vitro* differentiated derivatives including all 3 germ layers of ectoderm (NESTIN), mesoderm (alpha smooth muscle actin, α-SMA) and endoderm (GATA 4). Scale bar, 50 μm. **(D):** Teratoma (black arrow) formed 6 weeks after injection of riPS cells (LSP-24-24, passage 30) into SCID mouse. Hematoxylin-eosin (HE) stained sections of the teratoma showed all 3 germ layer derivatives; epidermis (ectoderm), cartilage (mesoderm) and intestinal epithelum (endoderm) are indicated by arrows. Scale bar, 100 μm. **(E):** Representative polyploid and diploid metaphase chromosome preps of riPS cells. 2i/Lif medium derived riPS clone showed a dominant polyploid metaphase chromosome while 3i/Lif medium derived riPS clone showed a dominant diploid metaphase chromosome.
